# Sinusoidal and pericellular fibrosis in adult post-transplant liver biopsies: association with hepatic stellate cell activation and patient outcome

**DOI:** 10.1007/s00428-019-02585-x

**Published:** 2019-06-14

**Authors:** Sameh Abou-Beih, Steven Masson, Rachael Saunders, Beate Haugk, Fiona Oakley, Dina Tiniakos

**Affiliations:** 1 Institute of Cellular Medicine, Faculty of Medical Sciences, Newcastle University, W. Leech Building, M4.143, Framlington Place, Newcastle upon Tyne, NE2 4HH UK; 2 Department of Pathology, Faculty of Medicine, Fayoum University, Fayoum, Egypt; 3 Liver Transplant Unit, Freeman Hospital, Newcastle upon Tyne, UK; 4 Department of Cellular Pathology, Royal Victoria Infirmary, NUTH NHS Trust, Newcastle upon Tyne, UK; 5 Department of Pathology, Aretaieion Hospital, Medical School, National & Kapodistrian University of Athens, Athens, Greece

**Keywords:** Liver fibrosis, Liver transplantation, Sonic hedgehog, Alpha-smooth muscle actin, Centrilobular fibrosis

## Abstract

Post-transplant sinusoidal fibrosis (SF) and pericellular fibrosis (PCF) have not been extensively investigated in adults. Fifty-two post-transplant liver biopsies from 28 consented patients (12 men, mean age 49, range 33–67 years) were studied. Tissue morphology, including an arbitrary summative fibrosis score was assessed in detail. Collagen proportionate area (CPA) and alpha-smooth muscle actin (α-SMA) immunostain were evaluated by digital image analysis (DIA). Anti-keratin 7, anti-C4d and anti-sonic hedgehog (Shh) immunostains were scored semi-quantitatively. SF was observed in 36/52 (69.2%) biopsies and most of these (20/36, 55.6%) had centrilobular fibrosis (CLF). PCF was seen in 7/52 (13.5%) biopsies exclusively in cases with CLF. CPA was significantly correlated with time since liver transplantation (*p* = 0.043), summative fibrosis score and its main components but not with α-SMA. α-SMA-positive area significantly correlated with the Banff rejection score (*p* = 0.022) and centrilobular inflammatory changes were more severe in cases with CLF (*p* = 0.003). Hepatocyte ballooning of cholestatic type was associated with PCF (*p* = 0.016) and Shh expression (*p* < 0.001). Sinusoidal fibrosis is a frequent occurrence in post-transplant adult livers, with predilection toward centrilobular areas. Graft age and oxidative stress may contribute to SF development, while hepatocyte ballooning may be implicated in PCF development. Hepatic stellate cell (HSC) activation is likely affected by centrilobular inflammation.

**Electronic supplementary material:**

The online version of this article (10.1007/s00428-019-02585-x) contains supplementary material, which is available to authorized users.

## Introduction

Sinusoidal fibrosis (SF) and pericellular fibrosis (PCF) are frequently observed in steatohepatitis of alcoholic and non-alcoholic aetiology [40]. They have also been reported as a frequent feature in the early phase of fibrosing cholestatic hepatitis C [10]. Post-transplant centrilobular fibrosis (CLF), most likely resulting from organisation of prior central perivenulitis lesions, has been mainly examined in paediatric recipients and chronic rejection has been implicated in its development [11, 14, 32, 42]. In adult post-transplant liver, periportal SF has been linked to recurrent hepatitis C [30]. Nevertheless, the significance of SF in adult post-transplant liver remains largely unexplored, while PCF in post-transplant liver has not previously been investigated.

Hepatic stellate cells (HSCs) are the main liver cells responsible for extra-cellular matrix deposition and their activation plays a role in the development of SF, CLF and PCF [3]. However, the role of HSCs has not been studied in detail in post-transplant liver [5, 16, 43] and their activation has mainly been assessed in association with fibrosis progression in recurrent hepatitis C [5, 16]. In non-alcoholic steatohepatitis (NASH), sonic hedgehog (Shh), a member of the hedgehog signalling pathway expressed in ballooned hepatocytes, is involved in HSC activation with subsequent PCF [44].

Studies evaluating the long-term outcome of the hepatic allograft have highlighted changes in the microvasculature, including sinusoidal dilation and SF [6, 12, 26]. Certain drugs are linked to SF development in the liver allograft, most notably azathioprine [6, 24]. Inadequate immunosuppression [11], preformed donor–specific antibodies [32] and chronic antibody–mediated rejection [6] have also been proposed as aetiological factors for SF in children. In the liver of renal transplant recipients, SF at the ultrastructural level has been linked to the development of portal hypertension [33].

We aimed to evaluate the frequency, extent and topography of SF and PCF in the adult post-transplant liver and its correlation with clinico-pathological features, HSC activation, ductular reaction (DR) and patient outcome.

## Materials and methods

### Patient population

This single-centre, retrospective cohort study was conducted at the Fibrosis Lab, Institute of Cellular Medicine, Newcastle University, and the Dept. of Cellular Pathology, Royal Victoria Infirmary, Newcastle upon Tyne, UK, on anonymised archival histological material from post-transplant liver biopsies obtained between March 2010 and September 2015 at Freeman Hospital, Newcastle upon Tyne, UK. During this period, 192 post-transplant liver specimens were obtained from 99 patients. Consent to use tissue for research was available for 29 patients with 59 liver specimens. Explant surgical specimens of failed allografts and biopsies without adequate amount of tissue material remaining in the paraffin block were excluded. Ethical approval was obtained from local ethical committee (Ethics approval number 10/H0906/41). No organs from executed prisoners were used in this study.

Applying the above-mentioned criteria, 52 core liver biopsies obtained where clinically indicated from 28 patients (12 men and 16 women), who had undergone a total of 29 liver transplantations, were available for the study. Mean age was 49.3 years (range 33–67) and median period of follow-up was 30 months (range 7–96). Median time to biopsy was 22 (interquartile range 9–35) days. Donors were 11 men, 18 women and one of unknown gender with average age at donation 51 years (range 24–80). Demographic details and results of liver function tests at time of biopsy, drug and medical history, indications of LT, presence and class of preformed donor–specific antibodies (DSA) and any other relevant details during follow-up were obtained from the hospital records and are summarised in Table 1.

**Table 1:** Summary of clinical details and laboratory results of the 28 study patients, 2 of which had undergone 2 liver transplantations (30 transplanted livers in total)

Features	
Indication for liver transplantation (*n* = 28)	*n* (%)
PBC	8 (28.6)
PSC	5 (17.9)
ALD	5 (17.9)
NASH/HCC	2 (7.1)
Drug related	2 (7.1)
Hepatitis (viral and autoimmune)	2 (7.1)
Surgical indications	3 (10.7)
Cryptogenic	1 (3.6)
Preformed donor–specific antibodies (*n* = 28)	
Present	6 (21.4)
Class I (A1, A3, B8)	4 (14.3)
Class II (DQ3, DQ7)	2 (7.1)
Absent	22 (78.6)
Complications and follow-up data (*n* = 28)	
HCC in the explant	4 (14.3)
Biliary complications	8 (28.6)
Vascular complications	9 (32.1)
Combined biliary and vascular complications	1 (3.6)
Post-operative sepsis	1 (3.6)
Allograft failure due to PSC recurrence	1 (3.6)
Death during follow-up	4 (14.3)
Cardiac causes	3 (10.7)
Acute pancreatitis	1 (3.6)
T2DM	4 (14.3)
Azathioprine therapy	18 (64.3)
Type of allograft (*n* = 29*)	
DBD	25 (86.2)
DCD	4 (13.8)
Allograft size (*n* = 29*)	
Whole liver	27 (93.1)
Reduced size (split)	2 (6.9)
Ischaemia duration in min (*n* = 29*)	*X̅* ± SD
Total cold ischaemia	580.03 ± 135.73
Recipient warm ischaemia	51.55 ± 16.79
Liver function tests at the time of biopsy	*X̅* ± SD
ALT (IU/L)	277.3 ± 293.04
Alkaline phosphatase (IU/L)	371.3 ± 240.08
Total bilirubin (μmol/l)	86.2 ± 103.87

Numbers and percentages are expressed in relation to number of available data as indicated by the *n* value beside each feature

*One patient out of the 28 included in the study underwent a 2nd liver transplantation due to failure of the 1st allograft (recurrent PSC)

*SD* standard deviation, *PBC* primary biliary cholangitis, *PSC* primary sclerosing cholangitis, *ALD* alcoholic liver disease, *NASH* non-alcoholic steatohepatitis, *HCC* hepatocellular carcinoma, *DSA* pre-transplant donor–specific HLA antibodies, *DBD* donation after brain death, *DCD* donation after circulatory death, *T2DM* type 2 diabetes mellitus

### Histopathologic assessment

Archival haematoxylin & eosin-stained slides were centrally reviewed by two histopathologists (SAB, DT). Sections were assessed for features of allograft rejection (including portal inflammation, bile duct injury, portal vessel endotheliitis, central perivenulitis and central vein endotheliitis) and graded using the Banff criteria [1], while interface hepatitis, lobular inflammation and confluent necrosis were assessed and graded according to the modified necro-inflammatory activity grading scheme [21]. All types of cholestasis (hepatocellular, canalicular and ductular) were graded 0–3 according to intensity and extent (0 = absent, 1 = mild, 2 = moderate, 3 = severe). Hepatocyte ballooning was scored 0–2 (0 = absent, 1 = few, 2 = many hepatocytes) [3]. Ischaemic changes (sinusoidal dilatation and congestion) were graded according to their extent (0 = absent, 1 = mild, 2 = moderate, 3 = severe). The presence of granulomas, mitotic figures and microabscesses was also assessed.

Sections, 4 μm thick, were cut from each block and stained with Sirius red fast green (SRFG) stain for the assessment of fibrosis and collagen proportionate area (CPA). Sinusoidal fibrosis (SF) was semi-quantitatively scored 0–2 (0 = absent, 1 = delicate SF, 2 = dense) to reflect the extent and thickness of sinusoidal fibrosis seen in adult liver allograft biopsies, which involved < 50% of sinusoids in the biopsies studied. Portal fibrosis and centrilobular fibrosis were scored (0–3) according to the histologic system proposed by Venturi et al. [41]. A summative fibrosis score was generated (0–8) by combining the three scores. PCF was diagnosed when collagen fibres surrounded individual hepatocytes or cords of hepatocytes in a “chicken-wire” pattern. PCF was assessed for presence and topography (centrilobular only, zones 3 & 2, panlobular). In this study, centrilobular fibrosis will refer to the mere presence of SF in centrilobular areas, regardless of its occurrence in other hepatic lobular zones, whereas isolated CLF will refer to SF restricted to zone 3.

### Immunohistochemical staining and assessment

A total of 4-μm-thick sections were immunostained for α-smooth muscle actin (α-SMA) to assess HSC activation and keratin 7 to assess DR and biliary metaplasia. For α-SMA immunostaining, sections were deparaffinised and rehydrated followed by standard endogenous peroxidase blocking. Heat-induced epitope retrieval (HIER) was performed using citrate-based antigen unmasking solution (Vector Laboratories Inc., Newcastle, UK) for 20 min. Avidin and biotin blocks were applied for 20 min each and a casein block was applied for 1 h (Vector Laboratories Inc., Newcastle, UK). The slides were then incubated for 1 h with a mouse monoclonal, anti-α-SMA antibody (FITC, Sigma-Aldrich, UK) diluted 1:3000 with 1% Casein block added. Slides were incubated for 2 h with biotinylated anti-fluorescein raised in goat, which was diluted 1:300 followed by incubation with ABC reagent, R.T.U. Vectastain kit (Vector Laboratories Inc., Newcastle, UK) for 1 h. Similar steps were used to immunostain sections for keratin 7 with a mouse monoclonal anti-human keratin 7 (clone OV-TL 12/30, Dako AS, Denmark) at 1:200 dilution at the automated immunostainer Ventana Benchmark®XT using the XT ultraView detection kit (Roche Diagnostics, USA). A subgroup of cases (*n* = 20) had been routinely immunostained for C4d with a rabbit monoclonal anti-C4d (clone A24-T) DB Biotech (Košice, Slovakia) at 1:200 dilution using the same automated method as above. Sections of cases with ballooned hepatocytes (*n* = 5) and of cases without ballooned hepatocytes used as controls (*n* = 4) were immunostained with a rabbit monoclonal antibody to sonic hedgehog (Shh) (anti-Shh ab53281, Αbcam, UK) at 1:3000 dilution with overnight incubation 4 °C using the Novolink™ Max Polymer Detection System (RE7280-K, Leica Biosystems Inc., UK). 3,3′-diaminobenzidine (DAB) was used as chromogen (Vector Laboratories Inc., Newcastle, UK). All slides were counterstained with Mayer’s Haematoxylin, dehydrated with alcohol, cleared in clearene and cover-slipped using Pertex (Histolab, Askim, Sweden).

Keratin 7 immunostained sections were assessed for the extent of ductular reaction (DR) using the scoring system devised by Gadd et al. [15] with slight modification (0–3, where 0 = absent, 1 = focal increased DR in portal tracts (PT) involving < 50% of PT circumference, 2 = DR in PT involving > 50% of PT circumference, 3 = distinct spurs of DR extending into the lobule). C4d immunostaining was assessed in the sinusoids, portal microvessels and portal stroma as absent, present when strong and diffuse or equivocal when mild and focal. Intralobular K7–positive cells with hepatic progenitor cell morphology, K7-positive hepatocytes and Shh-positive hepatocytes were semi-quantitatively scored 0–2 (0 = absent, 1 = few positive hepatocytes and 2 = many positive hepatocytes) and their acinar topography was recorded.

### Morphometric analysis for SRFG and α-SMA stains

Slides stained with SRFG and anti-α-SMA were photographed using a Nikon ECLIPSE Ni-U (Nikon UK Ltd) microscope. At least 15 optical fields were captured for SRFG and α-SMA-stained slides at × 10 magnification. CPA and percentage of α-SMA-stained area were quantified using Nikon Imaging Software Elements Basic Research (NIS-Elements BR Analysis 4.12.01, Nikon UK Ltd) and both were expressed as a percentage of parenchymal total area.

### Statistical methods

Values are expressed as percentages and measures of central tendency [mean (*X̅*), standard deviation (SD), median and range]. Chi square (*χ*^2^) test was used to compare categorical data. For comparison between 2 groups, Mann Whitney *U* test for non-parametric samples was used. Associations between variables were studied with non-parametric Spearman’s rank correlation coefficient (rho). Significant correlations were illustrated by means of linear regression. Stepwise multivariate regression analysis was used to identify most significant predictors of CPA and HSC activation. Correlations between allograft fibrosis, HSC activation parameters and duration of ischaemia were done by means of analysis of covariance (ANCOVA) with time since liver transplantation as the fixed variable. The probability (*p*) value was considered significant at a level < 0.05 and highly significant when *p* < 0.001. All statistical analyses were done using IBM SPSS Statistics software, version 23.0 (IBM Corp, Armonk, New York, USA).

## Results

### Histopathology

The median biopsy length was 18.5 mm (range 3–33 mm) and the median number of portal tracts per biopsy was 10 (range 3–24). The main histopathologic findings and diagnosis are described in Table 2. In all but one case, the clinical indication for liver biopsy was to investigate graft dysfunction. In the remaining case, liver biopsy was undertaken to help determine the need for antiviral therapy after LT for chronic hepatitis C.

**Table 2:** Detailed histopathological results in the studied liver biopsies (*n* = 52)

Feature	Present *n* (%)
Sinusoidal fibrosis	36 (69.2)
Delicate	26 (50)
Dense	10 (19.2)
Centrilobular fibrosis^1^	20 (38.5)
Mild	6 (11.5)
Moderate	12 (23.1)
Severe	2 (3.8)
Pericellular fibrosis	7 (13.5)
Centrilobular only	5 (9.6)
Zones 3 & 2	1 (1.9)
Panlobular	1 (1.9)
Acute cellular rejection (ACR)	38 (73.1)
Mild	7 (13.5)
Moderate	19 (36.5)
Severe	12 (23.1)
Portal inflammation	47 (90.4)
Mild	17 (32.7)
Moderate	15 (28.8)
Severe	15 (28.8)
Bile duct injury	43 (82.7)
Mild	21 (40.4)
Moderate	18 (34.6)
Severe	4 (7.7)
Portal endotheliitis	40 (76.9)
Mild	18 (34.6)
Moderate	13 (25)
Severe	9 (17.3)
Central perivenulitis	24 (46.2)
Mild	13 (25)
Moderate	10 (19.2)
Severe	1 (1.9)
Central vein endotheliitis	26 (50)
Mild	14 (29.6)
Moderate	5 (9.6)
Severe	7 (13.5)
Hepatocyte ballooning	5 (9.6)
Few	2 (3.8)
Many	3 (5.8)
Sinusoidal dilatation	39 (75)
Mild	35 (67.3)
Moderate	4 (7.7)
Severe	0 (0)
Sinusoidal congestion	29 (56.8)
Mild	27 (51.9)
Moderate	2 (3.8)
Severe	0 (0)
Lobular inflammation	50 (96.1)
Grade 1	13 (25)
Grade 2	23 (44.2)
Grade 3	13 (25)
Grade 4	1 (1.9)
Confluent necrosis	28 (53.8)
Grade 1	10 (19.2)
Grade 2	12 (23.1)
Grade 3	6 (11.5)
Grades 4–6	0 (0)
Interface hepatitis	18 (34.6)
Grade 1	15 (28.8)
Grade 2	2 (3.8)
Grade 3	1 (1.9)
Grade 4	0 (0)
Hepatocellular cholestasis	40 (76.9)
Mild	30 (57.7)
Moderate	8 (15.4)
Severe	2 (3.8)
Canalicular cholestasis	8 (15.4)
Mild	5 (9.6)
Moderate	2 (3.8)
Severe	1 (1.9)
Ductular cholestasis	1 (1.9)
Moderate	1 (1.9)
Portal fibrous expansion	32 (61.5)
Mild	18 (34.6)
Moderate	13 (25)
Severe	1 (1.9)
Ductular reaction extent	34 (65.4)
Mild	20 (38.5)
Moderate	7 (13.5)
Severe	7 (13.5)
Biliary metaplasia (K7+ hepatocytes)	14 (73.1)
Few	8 (15.4)
Many	6 (11.5)
K7+ cells with HPC morphology^2^	18 (34.6)
Few	17 (32.7)
Many	1 (1.9)
Other features	
Mitotic figures	7 (13.5)
Microabscesses	16 (30.8)
Granulomas	23 (44.2)
C4d immunostaining (*n* = 20)^3^	
Portal stroma	9 (45)
Portal microvessels	4 (20)
Sinusoids	4 (20)
Main histological diagnosis	
ACR	38 (73.1)
Ascending cholangitis	1 (1.9)
Recurrent hepatitis C	1 (1.9)
Preservation/reperfusion injury	1 (1.9)
Other and inconclusive diagnoses^4^	7 (13.5)
Venous outflow obstruction	4 (7.7)
Isolated	1 (1.9)
With ACR	1 (1.9)
With ductopenia	2 (3. 8)

*ACR* acute cellular rejection, *CPV* central perivenulitis, *CVE* central vein endotheliitis, *K7+* keratin 7-positive

^1^Only detected in biopsies showing SF; however, the ratios were given in relation to the total number of biopsies studied (*n* = 52)

^2^Intra-acinar K7+ cells with hepatic progenitor cell morphology

^3^The presence of C4d immunostaining was only assessed in a subgroup group of biopsies and percentages are given in relation to this subgroup (*n* = 20) and not the total number of biopsies (*n* = 52)

^4^These included inconclusive findings, cholestasis and granulomatous hepatitis

Thirty-six out of the 52 (69.2%) biopsies showed SF. Most of the biopsies with SF (20/36, 55.6%) exhibited CLF. In half of the cases with CLF (*n* = 10), this was restricted in zone 3. PCF was seen in 7/52 (13.5%) biopsies and only in those with CLF (7/20, 35%) (Table 2). PCF was centrilobular in five biopsies, involved zones 2 & 3 in one biopsy and was panlobular in one biopsy. SF, CLF and PCF were present in biopsies from 19 (67.9%), 13 (46.4%) and 5 patients (17.9%), respectively. Significant correlations between centrilobular inflammatory changes and histopathologic features of acute cellular rejection are shown in Supplementary Table S1.

### Immunohistochemical and morphometric results and their correlation with histological and clinical data

**Fig. 1:**
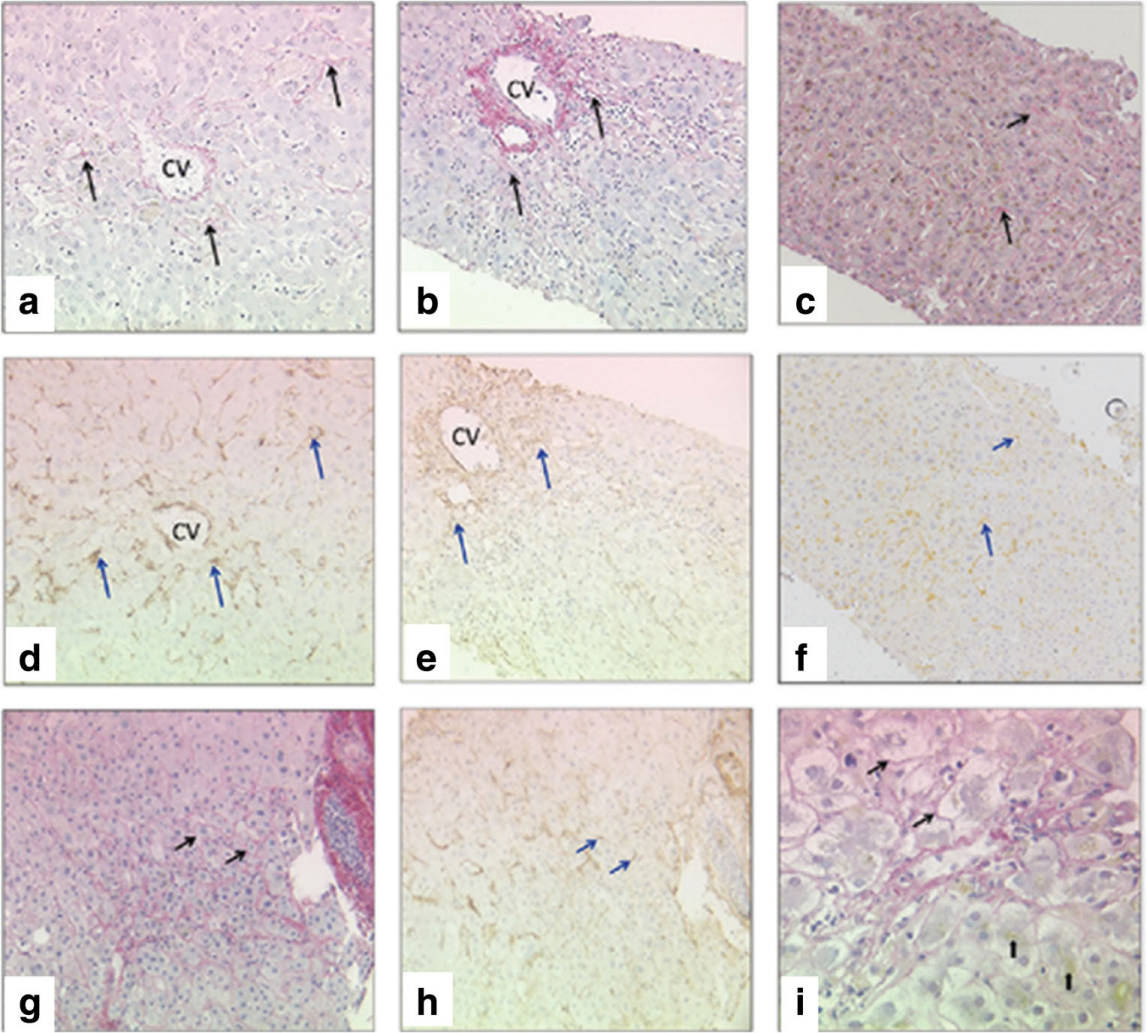
Serial sections of two cases with centrilobular fibrosis (**a**, **d** and **b**, **e**), a mild ACR case with non-zonal sinusoidal fibrosis (**c** and **f**) and a case with pericellular fibrosis (**G**-**I**): A, B, C & G. Sinusoidal collagen (black arrows, SRFG stain) and D-F, H. activated HSC (cyan arrows, α-SMA (immunohistochemical stain). I. Ballooned hepatocytes with associated fibrosis (arrows) in a case with canalicular cholestasis (arrowheads). Absence of fibrosis in relation to normal-sized hepatocytes (thick arrows) (SRFG), CV = central venule

The results of collagen proportionate area (CPA), percentage of α-SMA-stained area and summative fibrosis score are summarised in Table 3 along with significant correlations associated with each variable. Figure 1 shows representative images of cases with centrilobular fibrosis (a, d and b, e), sinusoidal fibrosis (c and f) and pericellular fibrosis (1G-I) stained with SRFG (a, b, c and g) or α-SMA (d–f, h).

**Table 3:** Significant correlations of collagen proportionate area, summative fibrosis score and α-SMA-stained area with results of multivariate analysis (most significant dependent variables) on the right side of the Table

Feature	Mean ± SD	Median (range)	Significant correlations			Multivariate analysis		
			Variable	rho	*p*	Dependent variable	Coefficient (*β*)	*p*
CPA	1.61 ± 2.384	0.66 (0.01–12.97)	Time since LT	0.282	0.043*			
			Summative fibrosis score	0.49	< 0.001**	(intercept)	0.7177	
			CLF grade	0.324	0.019*	Time since LT	0.002782	0.0001**
			SF extent	0.504	< 0.001**	CLF grade	0.7665	0.01*
Summative fibrosis score	2.46 ± 1.841	2 (0–8)	Time since LT	0.341	0.013*			
			CPA	0.49	< 0.001**	(intercept)	0.2134	
			Biliary metaplasia	0.346	0.012*	CLF grade	0.9726	< 0.0001**
			DR grade	0.336	0.015*	SF extent	1.2771	< 0.0001**
			CLF grade	0.591	< 0.001**	DR grade	0.4390	< 0.0001**
			SF extent	0.801	< 0.001**			
			Portal fibrosis	0.753	< 0.001**			
α-SMA%	1.54 ± 1.301	1.07 (0–4.71)	CLF grade	0.32	0.021*			
			RAI	0.318	0.022*	(intercept)	1.0536	
			CPV	0.331	0.017*	Confluent necrosis	0.4843	0.003*
			CVE	0.370	0.007*			
			Confluent necrosis	0.519	< 0.001**			

*CPA* collagen proportionate area, *α-SMA%* percentage of α-SMA-stained areas, *CLF* centrilobular fibrosis, *SF* sinusoidal fibrosis, *DR* ductular reaction, *RAI* rejection activity index (BANFF score), *CPV* central perivenulitis, *CVE* central vein endotheliitis, *LT* liver transplantation, *rho* correlation coefficient

*Significant

**Highly significant

**Fig. 2:**
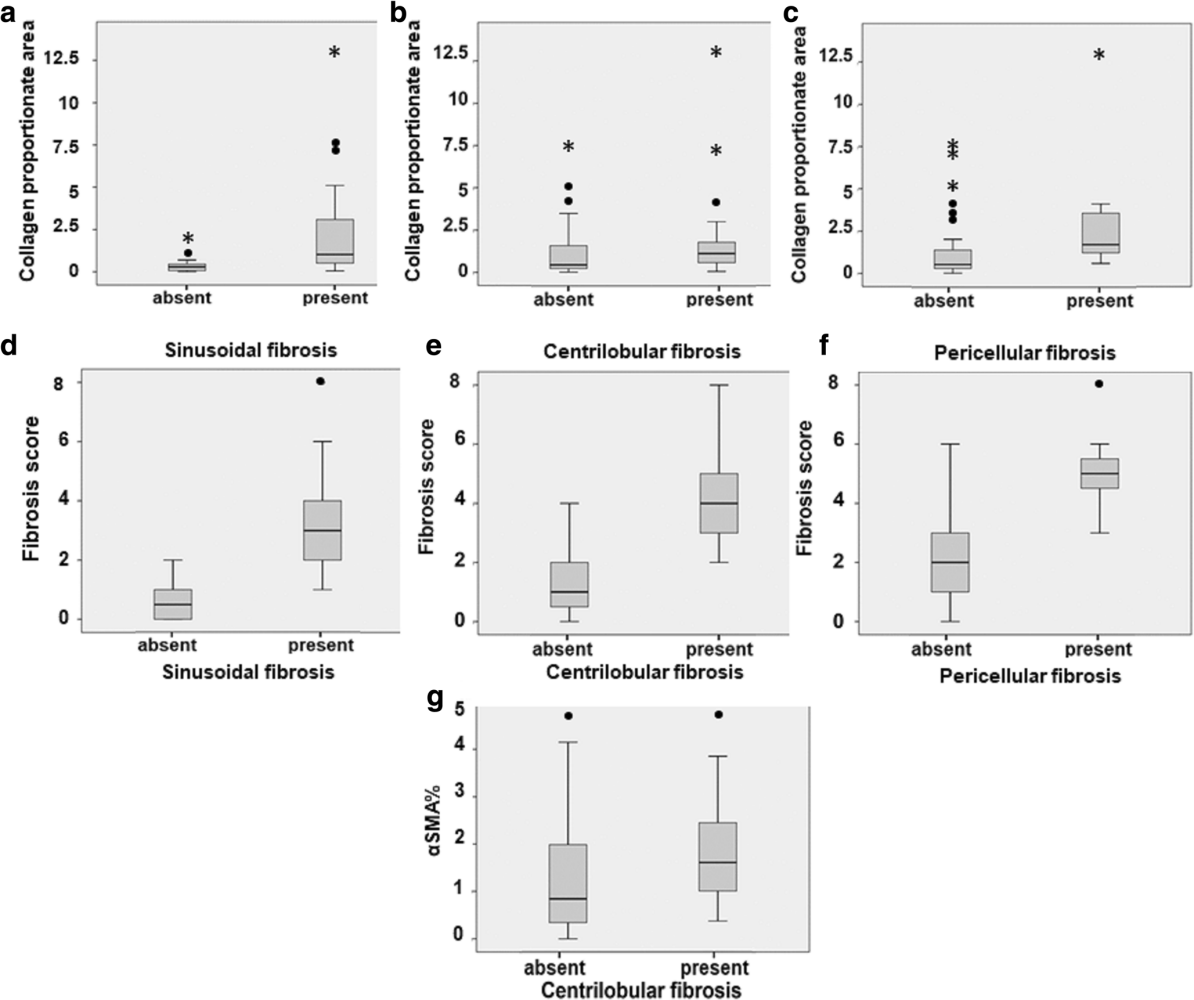
Graphs show correlations between measures of allograft fibrosis [summative fibrosis score and collagen proportionate area (CPA)] and hepatic stellate cell activation (α-SMA%) with patterns of allograft fibrosis in parenchyma [sinusoidal fibrosis (SF), centrilobular fibrosis (CLF) and pericellular fibrosis (PCF)], in study cohorts (*n* = 52). Data are presented as medians and quartile intervals. **a**–**c** CPA was higher in cases with SF (**a**), CLF (**b**) and PCF (**c**). **d**–**f** Summative fibrosis score was higher in cases with SF (**d**), CLF (**e**) and PCF (**f**). **g** α-SMA% was higher only in cases exhibiting CLF

Summative fibrosis score and CPA were significantly higher in cases with SF (*p* < 0.001 for both), CLF (*p* < 0.001 and *p* = 0.031, respectively) and PCF (*p* < 0.001 and *p* = 0.015, respectively) (Fig. 2a–f). α-SMA-positive area was only significantly higher in cases with CLF (*p* = 0.044) (Fig. 2g). Neither CPA nor summative fibrosis score had any significant correlation with α-SMA-positive area. Cases exhibiting SF without CLF did not show any statistically significant differences in CPA, summative fibrosis score or α-SMA-positive area when compared with cases showing CLF in association with parenchymal SF (*p* = 0.149, 0.559 and 0.372, respectively). CLF was significantly correlated with confluent necrosis (*p* = 0.003).

**Fig. 3:**
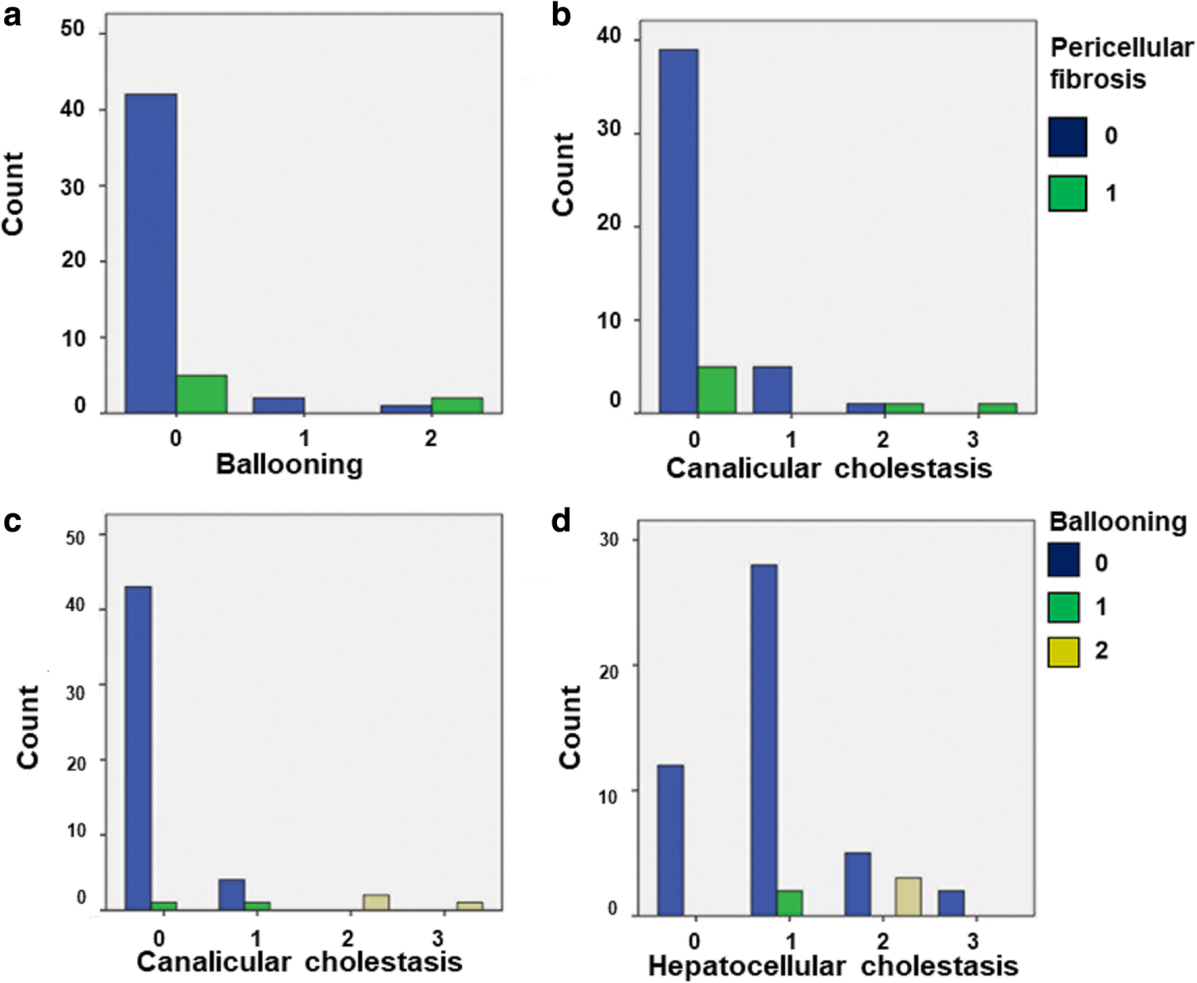
Significant associations of pericellular fibrosis (PCF) and hepatocyte ballooning of cholestatic origin. **a** and **b** PCF was significantly associated with ballooning and canalicular cholestasis. **c** and **d** Ballooning significantly associated with canalicular and hepatocellular cholestasis

**Fig. 4:**
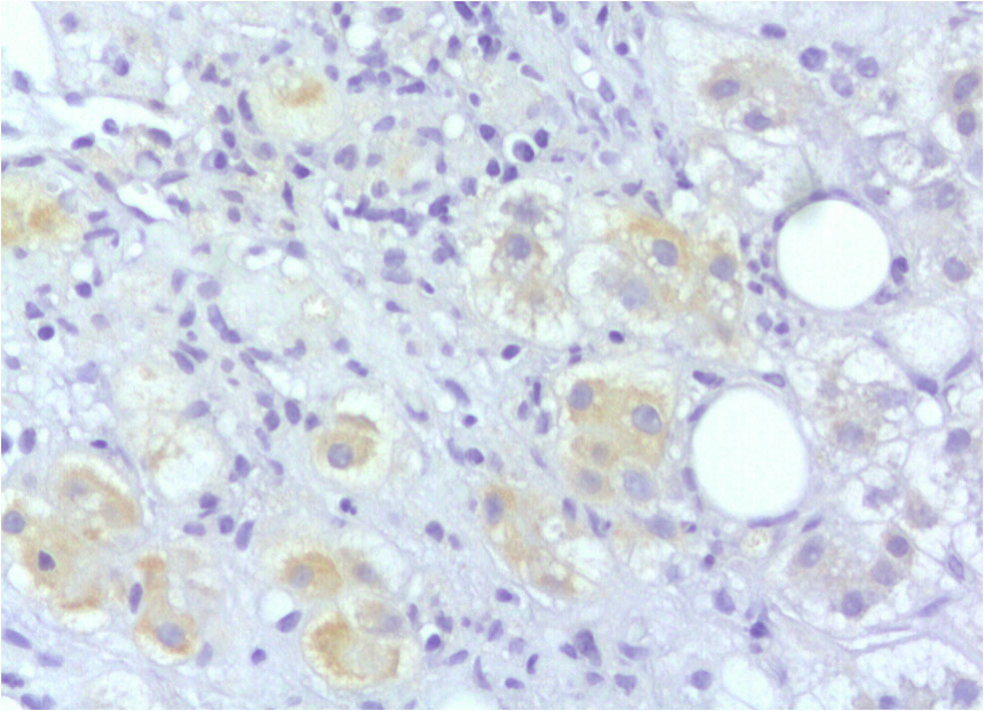
Shh immunostaining in a case with severe ACR and cholestasis (×200)

A significant association was found between hepatocyte ballooning and canalicular cholestasis (*p* < 0.001), hepatocellular cholestasis (*p* = − 0.004) and PCF (*p* = 0.019) (Fig. 3a, c, d). Shh immunohistochemical expression (Fig. 4) was significantly associated with hepatocyte ballooning (*p* < 0.001), the presence of DR (*p* = 0.02), SF (*p* = 0.036) and hepatocellular cholestasis (*p* = 0.02). A significant association was also found between PCF and canalicular cholestasis (*p* = 0.022), indicating a correlation between PCF and hepatocellular ballooning of cholestatic origin (Fig. 3a, b). Sinusoidal congestion alone was associated with less hepatocyte ballooning (*p* = 0.031); however, neither sinusoidal congestion nor dilatation had any significant association with PCF. CLF and PCF were associated with longer time since liver transplantation (*p* = 0.014 and *p* = 0.043, respectively).

Portal fibrous expansion score showed a highly significant linear correlation with DR grade (*p* < 0.001), a significant correlation with biliary metaplasia and an inverse linear correlation with interlobular keratin 7–positive cells with hepatic progenitor cell morphology. C4d immunostaining results (sinusoidal, portal microvascular or portal stromal) were available for 20 cases and there was no significant association with SF, CLF or PCF independent of topography.

Multivariate regression analysis highlighted confluent necrosis as the most significant predictor for HSC activation (assessed by α-SMA-positive area), whereas time since liver transplantation proved to be the most significant predictor of CPA followed by CLF grade. The most significant predictors of the summative fibrosis score were CLF grade, SF extent and DR score (Table 3).

SF, PCF and α-SMA-positive area scores were independent of patient age or gender, donor’s age or gender, indication for liver transplantation, liver function test values, rejection type, pre-transplant donor–specific antibodies or biliary/vascular complications.

Almost half of biopsies (17 biopsies, 47.2%), exhibiting SF, were receiving azathioprine at the time of biopsy, compared to 6 biopsies only (37.5%), who did not exhibit SF, with no statistically significant differences (odds ratio 1.5741, *p* = 0.462). Similarly, mycophenolate mofetil (MMF) was administered by 44.4% (*n* = 16) of biopsies showing SF, and 56.25% (*n* = 9) of SF-negative biopsies (odds ratio 0.7778, *p* = 0.638). Administration of both medications does not appear to be associated with any statistically significant difference in summative fibrosis score, CPA or α-SMA-positive area.

Duration of ischaemia did not correlate with liver allograft fibrosis markers (summative fibrosis score and CPA) nor with α-SMA-positive area; however, cases exhibiting PCF were associated with longer warm ischaemia duration (*X̅* = 69.14 vs 49.8 min, *p* = 0.009), and so did CLF-positive cases (*X̅* = 57.3 vs 49.26 min, *p* = 0.05).

## Discussion

To our knowledge, this is the first study to extensively investigate sinusoidal fibrosis (SF) and pericellular fibrosis (PCF) and their relation to hepatic stellate cell activation in adult post-transplant livers. We showed that SF is a common finding in adult post-transplant livers, existing in more than two thirds of the studied biopsies and had a predilection for centrilobular areas. Although SF has previously been reported in a variety of liver disorders, post-transplant SF was rarely mentioned in adult post-transplant liver: periportal SF in the context of recurrent hepatitis C has been reported as a marker of accelerated post-transplant recurrence [30], and as one of the suggested criteria for diagnosing fibrosing cholestatic hepatitis C [45]. In contrast, SF has been extensively studied in paediatric post-transplant liver where the presence of CLF ranged from 10.3 [31] to 95%; however, isolated CLF was seen in only 5% of the cases in the latter series [42]. In our study, isolated CLF is much more prevalent (50% of biopsies exhibiting CLF).

In fact, CLF appears to be a more progressive form of SF, as supported by our finding of longer time since liver transplantation (LT) in cases with CLF. This is re-enforced by the strong correlation between time since LT and both CPA and summative fibrosis score and the fact that time since LT and CLF severity were the most significant predictors of CPA in our study. CPA is significantly correlated with hepatic venous pressure gradient, used as a measure of portal hypertension [20, 28, 29]. Ling et al. [27] have reported results supportive of such theory with portal hypertension–related complications identified by imaging in almost half of the paediatric population in their study. Indeed, progressive graft fibrosis occurs in paediatric recipients, [13, 36] with cirrhosis developing in 15% of the cases [13]. Along these lines, Venturi et al. showed increasing liver allograft fibrosis score (LAFSc) with time in paediatric transplant biopsies. Our results showing timely progression of allograft fibrosis (by both the summative fibrosis score and CPA) suggest that similar changes are likely to take place in adult recipients indicating that longer period of follow-up may be advisable.

Sinusoidal fibrosis has been described in ageing animal [18] and human [25] livers. Centrilobular SF is the characteristic pattern of early fibrosis in alcoholic steatohepatitis (ASH) and adult NASH. The effects of reactive oxygen species (ROS) and oxidative stress underlie the pathogenesis of SF in NASH [34, 39] and ageing liver [8, 37]. Replicative senescence resultant in telomere attrition is the classic theory of ageing [4, 22]. Both mechanisms appear to be intertwined [7]. Liver cells are susceptible to senescence and stress-related senescence can be accelerated by the stress of transplantation. Cellular senescence is suggested to be accountable for some of the long-term deterioration and damage of liver allografts [19]. Aini et al. [2] demonstrated telomere shortening in transplanted livers of paediatric recipients.

A vascular aetiology for the development of SF in post-transplant liver cannot be excluded. Features of venous outflow obstruction have been reported in the right lobe of live donor transplanted livers after volume restoration [38]. Mild SF related to hepatocyte atrophy may be seen in nodular regenerative hyperplasia (NRH), whereas fibrosis typical of chronic liver disease is usually absent [35]. SF could also reflect changes in sinusoidal blood flow, similar to what occurs in NRH. NRH may be seen de novo in post-transplant biopsies [9].

Only three studies have investigated the role of activated HSC in post-transplant livers to date [5, 16, 42], two of which focused on recurrent hepatitis C. Gawrieh et al. [16] concluded that HSC activation could predict progression of fibrosis in recurrent HCV infection. Cisneros et al. [5] reported a correlation between HSC activation and rate of fibrosis progression in patients with recurrent HCV post-transplant; however, they did not show any difference in HSCs activation and accelerated fibrosis as compared to non-LT patients with chronic hepatitis C. Venturi et al. [42] reported that ≥ 8% activated HSCs (α-SMA%) at 6-month post-transplant in paediatric patients is an independent risk factor for 7-year fibrosis development and that α-SMA% ≥ 8% at 3-year post-transplant is associated with fibrosis development at 7 years. None of these studies investigated HSCs correlation with SF or CLF. However, in our study with only one HCV–infected patient, α-SMA% has not exceeded 4.71% and we did not encounter any correlation between α-SMA% and allograft fibrosis parameters apart from that with CLF. Conversely, α-SMA% was highly significantly correlated with centrilobular inflammatory changes, either involving central veins or adjacent hepatocytes. Activated HSCs, as well as being a key player in fibrogenesis, also help to activate and promote hepatic epithelial cell regeneration. A variety of stimulants including growth factors and cytokines are involved in the process of HSC activation in response to hepatic injury [46]. Centrilobular hepatocytes expressing glutamine synthetase were shown to be the last cells to proliferate in the process of liver regeneration [17], as well as being the most vulnerable to oxidative stress due to their poor oxygen supply. This may explain the predilection of SF to centrilobular regions, as the delayed proliferation of these cells, in the setting of activated HSCs, may allow more collagen fibres to be deposited in centrilobular regions during healing.

PCF affecting perivenular (zone 3) hepatocytes, also known as “chicken-wire” fibrosis, is typically seen in both ASH and NASH [40]. We have assessed for the first time PCF in adult post-transplant liver and highlighted a significant association between its presence and hepatocyte ballooning of cholestatic aetiology. In addition, ballooned hepatocytes in all cases were positive for Shh, including the two biopsies with PCF. The correlation of Shh expression with SF in our small cohort can be explained by the role of hedgehog signalling pathway in HSC activation. The recently exposed role of HSC activation in the metabolic zonation of the liver may also underlie the predilection of PCF in zone 3 in our study as well as in other liver disorders [23]. Our study is the first to show that Shh is expressed in ballooned hepatocytes of cholestatic aetiology.

The main limitations of our study include the lack of protocol biopsies, very small number of recipients of reduced-sized grafts and very small number of recurrent hepatitis C cases. However, ours was a single-centre study and we followed a non-biased approach in our sampling protocol. Regardless of these limitations, we have demonstrated the frequent presence of SF in adult post-transplant liver and its predilection to centrilobular areas. We could not show any short-term effects of SF or CLF on the liver allograft; however, our results support a progressive development of these fibrotic processes. A longer observation period may highlight possible long-term effects of SF and CLF. Moreover, while PCF was not a common finding, we showed a correlation of PCF with Shh-expressing ballooned hepatocytes indicating a possible involvement of the Shh pathway in the pathogenesis of PCF in the liver allograft that warrants further in-depth investigation at the molecular level.

## Electronic supplementary material

ESM 1 (DOCX 26 kb)

## Abbreviations

ASH: Alcoholic steatohepatitis
α-SMA: Alpha-smooth muscle actin
CPV: Central perivenulitis
CVE: Central vein endotheliitis
CLF: Centrilobular fibrosis
CPA: Collagen proportionate area
DIA: Digital image analysis
DSAs: Donor-specific antibodies
DR: Ductular reaction
HCV: Hepatitis C virus
HSCs: Hepatic stellate cells
K7: Keratin 7
LT: Liver transplantation
NASH: Non-alcoholic steatohepatitis
NRH: Nodular regenerative hyperplasia
MMF: Mycophenolate mofetil
PCF: Pericellular fibrosis
PT: Portal tracts
ROS: Reactive oxygen species
SF: Sinusoidal fibrosis
Shh: Sonic hedgehog
