# Supplementary material for: Sinusoidal and pericellular fibrosis in adult post-transplant liver biopsies: association with hepatic stellate cell activation and patient outcome
Source: Virchows Arch. 2019 Jun 14;475(2):233–43. doi: 10.1007/s00428-019-02585-x (PMC6647882; doi:10.1007/s00428-019-02585-x)
Supplement: Supplementary file 1 — (DOCX 26 kb) [file 428_2019_2585_MOESM1_ESM.docx]

**Supplementary material.**

**Table S1.** Significant correlations between centrilobular inflammatory changes and histopathologic features of acute cellular rejection.

| Centrilobular lesion | Associated histopathologic observation | *rho* | *P* |
| --- | --- | --- | --- |
| Central Perivenulitis (CPV) | CVE | 0.794 | 0.001^**^ |
|  | Rejection Activity index (RAI) | 0.542 | 0.001^**^ |
|  | Portal endotheliitis | 0.634 | 0.001^**^ |
|  | Portal inflammation | 0.42 | 0.002^*^ |
|  | Bile duct injury | 0.286 | 0.04^*^ |
| Central vein endotheliitis (CVE) | CPV | 0.794 | 0.001^**^ |
|  | RAI | 0.681 | 0.001^**^ |
|  | Portal endotheliitis | 0.766 | 0.001^**^ |
|  | Portal inflammation | 0.525 | 0.001^**^ |
|  | Bile duct injury | 0.426 | 0.002^*^ |

CPV: Central Perivenulitis, CVE: central vein endotheliitis., RAI: rejection activity index (Banff score), rho: correlation coefficient.

* significant, ** highly significant
